# Positively Charged Electroceutical Spun Chitosan Nanofibers Can Protect Health Care Providers From COVID-19 Infection: An Opinion

**DOI:** 10.3389/fbioe.2020.00885

**Published:** 2020-08-18

**Authors:** Rania M. Hathout, Dina H. Kassem

**Affiliations:** ^1^Department of Pharmaceutics and Industrial Pharmacy, Faculty of Pharmacy, Ain Shams University, Cairo, Egypt; ^2^Department of Biochemistry, Faculty of Pharmacy, Ain Shams University, Cairo, Egypt

**Keywords:** nanofi bers, COVID - 19, health care provider (HCP), electrospinning, SARS - CoV-2, textile–clothing industry

## Introduction

Corona virus infectious disease 19 (COVID-19) is a seriously alarming pandemic (https://www.who.int/emergencies/diseases/novel-coronavirus-2019). As of 13th June 2020, about 7,495,164 confirmed cases and 421,976 confirmed deaths have been reported globally by the World health Organization (WHO) (https://www.who.int/emergencies/diseases/novel-coronavirus-2019). During this crisis, health care providers (HCPs) are at the front line, exerting enormous efforts and facing great challenges in the battle to fight COVID-19 (Xiong and Peng, 2020). An interesting study in The Lancet Global Health journal, by Qian Liu and colleagues, highlighted the experiences of HCPs during the COVID-19 crisis in China, which by the way also provides a picture of the challenges faced by the HCPs all over the world during the COVID-19 outbreak. In that study, the authors emphasized that the high risk of infection and insufficient personal protective equipment are among the main causes of enormous pressure on HCPs during the outbreak (Liu et al., 2020). Undoubtedly, safety of HCPs is of crucial importance, and ensuring their safety will ensure efficacy in crisis management and COVID-19 containment.

The severe acute respiratory syndrome corona virus 2 (SARS-CoV-2) is an enveloped positive-stranded RNA virus which causes COVID-19 (Gorbalenya et al., 2020). In fact, breathing and talking of an infected person, produce numerous aerosol particles which pose a threat of infection if they are inhaled by nearby close persons (Meselson, 2020). Moreover, a contact hazard usually develops since these particles might settle on surfaces, and remain viable/infective for several hours (van Doremalen et al., 2020). In other cases, these particles could be too small to settle and remain dispersed by air turbulence, posing an inhalation threat even at considerable distances (Meselson, 2020). Consequently, these observations highlight the crucial importance of providing adequate personal protective equipment and clothes especially for the HCPs while taking care of COVID-19 patients.

Careful consideration for the structural features of SARS-CoV-2 will provide insights for better therapeutic as well as protective measures to combat COVID-19. Basically, the SARS-CoV-2 has four major structural proteins: the spike (S) protein, the envelope (E) protein, membrane (M) protein, and the nucleocapsid (N) protein (Schoeman and Fielding, 2019; Kang et al., 2020; Ou et al., 2020). Like other RNA viruses, SARS-CoV-2 consists of a negatively charged RNA enveloped inside a positively charged capsid that holds this genetic material firmly and plays a very important role in the virus infectivity (Belyi and Muthukumar, 2006; Hu et al., 2008; Forrey and Muthukumar, 2009).

Knowing that the particle size of the virus was reported to be in the range of 70–90 nm (Kim et al., 2020). Therefore, SARS-CoV-2 is considered a colloidal particle and a nanoparticle, specifically, and hence likewise all the colloidal particles, it carries a zeta-potential which in this case as previously mentioned is mostly assumed to be a positive one, primarily due to its capsid (Forrey and Muthukumar, 2009).

Luckily, the previous knowledge about corona-viruses helped the scientific community to achieve rapid progress in understanding SARS-CoV-2, however much remains unknown, and its biology is still far from complete elucidation and new discoveries and findings are emerging every day (Andersen et al., 2020; Gorbalenya et al., 2020). For example, it's noteworthy in this context to mention that novel polybasic (arginine rich) motifs, imparting a positive zeta potential at the physiological pH, have been identified in the spike protein of SARS-CoV-2, which seems to be a distinguishing unique feature compared to several previous SARS-related sequences (Jaimes et al., 2020). These motifs were reported to play important role during the process of SARS-CoV-2 entrance to host cells together with implications on virus infectivity (Hoffmann et al., 2020; Jaimes et al., 2020).

In this context, we introduce an opinion that could serve health-care providers by exploiting positively charged polymers such as chitosan in order to prepare nanofibers possessing a positive zeta-potential which can be incorporated in the personal protective clothes (fabrics) of the health-care providers and could lead to the electrostatic virus repulsion and hence decrease the viral load around those health-care providers.

The produced nanofibers are either directly electrospun into membranes or fabrics or can be integrated in protective clothes by physical adsorption and adhesion to the clothes material thanks to their very high surface area (Baji et al., 2020).

## Why Chitosan?

Chitosan is a natural cationic polysaccharide that exhibits many remarkable properties such as biocompatibility, biodegradability, non-toxicity, hemostatic and bio-adhesiveness and penetration enhancing properties (Abdel-Hafez et al., 2014, 2018). Moreover, chitosan has remarkable anti-microbial properties in addition to being abundant and in-expensive (Qi et al., 2004; Abdel-Hafez et al., 2018). It is worth noting, that quaternized chitosan such as the oldest form N,N,N-trimethyl chitosan (TMC) or the most recently prepared derivatives; the single *N*-quaternized (QCS) and the double *N*-diquaternized (DQCS) chitosan derivatives, possessing more positively charged amino groups can give better viral repulsion results. However, for large-scale production purposes, we propose to use chitosan due to its wide-abundance and availability rather than its derivatives that warrant long chemical synthesis schemes for their production (Farid et al., 2014; El-Marakby et al., 2017; Luan et al., 2018; Abdelhamid et al., 2019).

## Why Electrospinning?

The electrospinning technique provides non-wovens to the order of few nanometers with large surface areas, ease of functionalization for various purposes and superior mechanical properties. Also, the possibility of large scale productions combined with the simplicity of the process makes this technique very attractive for many different applications. The biomedical field is one of the important application areas among others utilizing the technique of electrospinning whether for drug delivery or for protection or prophlaxis purposes (Agarwal et al., 2008).

## Why Nanofibers?

Electrospinning has several merits of simplicity, high efficiency, low cost, and high reproducibility. Electrospinning was first invented as a patent to produce continuous fibers in 1934. Since this date, high attention was given to this valuable technique and its applications. Compared to conventional fibrous structures, nanofibers are lightweight with small diameters in the nano range, controllable pore structures and high surface-to-volume ratio. These remarkable properties make them ideal for use in a wide array of applications such as filtration, sensors, protective clothing, tissue engineering, functional materials, and energy storage (Cai et al., 2012).

## Proposed Method of the Chitosan Nanofibers Incorporation Into Textiles and Fabrics

A straight forward and a simple approach of incorporating electrospun fibers into textile involves electrospinning the fibers directly on the surface of the textile or fabric to obtain a composite fabric. This approach of depositing fibers on the surface of the fabric is considered highly economic and reduces the number of manufacturing steps and therefore is appropriate for mass production (Vitchuli et al., 2010). Increasing the bonding efficiency of the deposited nanofibers and the fabrics or textiles can be achieved through several techniques whether thermal, through the deposition of several dense layers, needling and hydro-entanglement or through the use of an adhesive (Midha and Dakuri, 2017). Furthermore, the electrospun chitosan nanofibers were successfully mass produced and its large-scale production was previously reported through a Force spinning technology® (Zhang et al., 2008; Xu et al., 2014).

**Figure 1:**
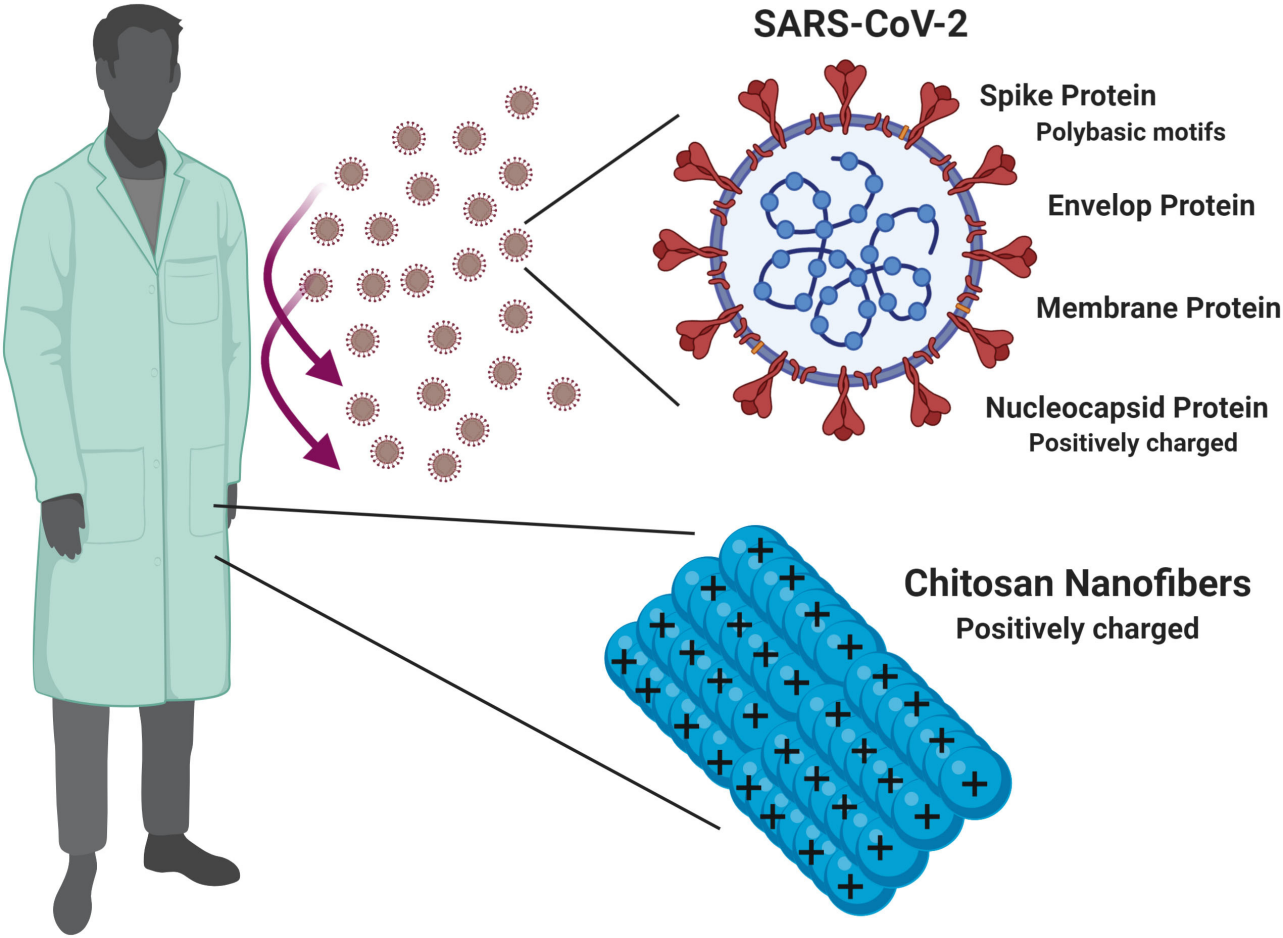
A schematic presentation for the idea of incorporating positively charged chitosan nanofibers in the personal protective clothes/fabrics of the health care providers, and how such modality can help to decrease the viral load around the health care providers, and hence enhance their protection, and safety during the current COVID-19 crisis.

Figure 1 presents a schematic summary of the proposed idea.

## Author Contributions

RH: conceptualization, hypothesis, methodology, discussion, writing, and revision. DK: methodology, discussion, writing, and revision. All authors: contributed to the article and approved the submitted version.

## Conflict of Interest

The authors declare that the research was conducted in the absence of any commercial or financial relationships that could be construed as a potential conflict of interest.

## References

[B1] Abdel-Hafez S. M. Hathout R. M. Sammour O. A. (2014). Towards better modeling of chitosan nanoparticles production: screening different factors and comparing two experimental designs. Int. J. Biol. Macromol. 64, 334–340. 10.1016/j.ijbiomac.2013.11.041 24355618

[B2] Abdel-Hafez S. M. Hathout R. M. Sammour O. A. (2018). Tracking the transdermal penetration pathways of optimized curcumin-loaded chitosan nanoparticles via confocal laser scanning microscopy. Int. J. Biol. Macromol. 108, 753–764. 10.1016/j.ijbiomac.2017.10.170 29104049

[B3] Abdelhamid H. N. El-Bery H. M. Metwally A. A. Elshazly M. Hathout R. M. (2019). Synthesis of CdS-modified chitosan quantum dots for the drug delivery of Sesamol. Carbohydr. Polym. 214, 90–99. 10.1016/j.carbpol.2019.03.024 30926012

[B4] Agarwal S. Wendorff J. H Greiner A. (2008). Use of electrospinning technique for biomedical applications, Polymer 49, 5603–5621. 10.1016/j.polymer.2008.09.014

[B5] Andersen K. G. Rambaut A. Lipkin W. I. Holmes E. Garry R. F. (2020). The proximal origin of SARS-CoV-2. Nat. Med. 26, 450–452. 10.1038/s41591-020-0820-9 32284615 PMC7095063

[B6] Baji A. Agarwal K. Oopath S. V. (2020). Emerging developments in the use of electrospun fibers and membranes for protective clothing applications. Polymers 12:492. 10.3390/polym12020492 32102318 PMC7077639

[B7] Belyi V. A. Muthukumar M. (2006). Electrostatic origin of the genome packing in viruses. Proc. Natl. Acad. Sci. U.S.A. 103:17174. 10.1073/pnas.0608311103 17090672 PMC1859905

[B8] Cai Y. Wei Q. Huang F. (2012). 3 - Processing of composite functional nanofibers, in Functional Nanofibers and their Applications Woodhead Publishing Series in Textiles, eds Wei Q.. (Cambridge: Woodhead Publishing), 38–54.

[B9] El-Marakby E. M. Hathout R. M. Taha I. Mansour S. Mortada N. D. (2017). A novel serum-stable liver targeted cytotoxic system using valerate-conjugated chitosan nanoparticles surface decorated with glycyrrhizin. Int. J. Pharm. 525, 123–138. 10.1016/j.ijpharm.2017.03.081 28392279

[B10] Farid M. M. Hathout R. M. Fawzy M. bou-Aisha K. (2014). Silencing of the scavenger receptor (Class B - Type 1) gene using siRNA-loaded chitosan nanaoparticles in a HepG2 cell model. Colloids Surf. B Biointerfaces 123, 930–937. 10.1016/j.colsurfb.2014.10.045 25466457

[B11] Forrey C. Muthukumar M. (2009). Electrostatics of capsid-induced viral RNA organization. J. Chem. Phys. 131:105101 10.1063/1.3216550

[B12] Gorbalenya A. E. Baker S. C. Baric R. S. de Groot R. J. Drosten C. Gulyaeva A. A.. (2020). The species Severe acute respiratory syndrome-related coronavirus: classifying 2019-nCoV and naming it SARS-CoV-2. Nat. Microbiol. 5, 536–544. 10.1038/s41564-020-0695-z 32123347 PMC7095448

[B13] Hoffmann M. Kleine-Weber H. Pohlmann S. (2020). A multibasic cleavage site in the spike protein of SARS-CoV-2 is essential for infection of human lung cells. Mol. Cell 78, 779–784. 10.1016/j.molcel.2020.04.022 32362314 PMC7194065

[B14] Hu T. Zhang R. Shklovskii B. I (2008). Electrostatic theory of viral self-assembly. Phys. A 387, 3059–3064. 10.1016/j.physa.2008.01.010

[B15] Jaimes J. A. Millet J. K. Whittaker G. R. (2020). Proteolytic cleavage of the SARS-CoV-2 spike protein and the role of the novel S1/S2 site. iScience 23:101212. 10.1016/j.isci.2020.101212 32512386 PMC7255728

[B16] Kang S. Yang M. Hong Z. Zhang L. Huang Z. Chen X.. (2020). Crystal structure of SARS-CoV-2 nucleocapsid protein RNA binding domain reveals potential unique drug targeting sites. Acta Pharm. Sin. B. 10.1016/j.apsb.2020.04.009 32363136 PMC7194921

[B17] Kim J. M. Chung Y. S. Jo H. J. Lee N.-J Kim M. S. Woo S. H.. (2020). Identification of Coronavirus Isolated from a Patient in Korea with COVID-19. Osong Public Health Res. Perspect 11 3–7. 10.24171/j.phrp.2020.11.1.02 32149036 PMC7045880

[B18] Liu Q. Luo D. Haase J. E. Guo Q. Wang X. Liu S.. (2020). The experiences of health-care providers during the COVID-19 crisis in China: a qualitative study. Lancet Global Health 8, e790–e798. 10.1016/S2214-109X(20)30204-7 32573443 PMC7190296

[B19] Luan F. Wei L. Zhang J. Tan W. Chen Y. Dong F.. (2018). Preparation and characterization of quaternized chitosan derivatives and assessment of their antioxidant activity. Molecules 23:516. 10.3390/molecules23030516 29495379 PMC6017865

[B20] Meselson M. (2020). Droplets and aerosols in the transmission of SARS-CoV-2. N. Engl. J. Med. 382:2063. 10.1056/NEJMc2009324 32294374 PMC7179963

[B21] Midha V. K. Dakuri A. (2017). Spun bonding technology and fabric properties: a review. J. Text. Eng. Fash. Technol. 1, 126–133. 10.15406/jteft.2017.01.00023

[B22] Ou X. Liu Y. Lei X. Li P. Mi D. Ren L.. (2020). Characterization of spike glycoprotein of SARS-CoV-2 on virus entry and its immune cross-reactivity with SARS-CoV. Nat. Commun. 11:1620. 10.1038/s41467-020-15562-9 32221306 PMC7100515

[B23] Qi L. F. Xu Z. R. Jiang X. Hu C. Zou X. (2004). Preparation and antibacterial activity of chitosan nanoparticles. Carbohydrate Res. 339, 2693–2700. 10.1016/j.carres.2004.09.007 15519328

[B24] Schoeman D. Fielding B. C. (2019). Coronavirus envelope protein: current knowledge. Virol. J. 16:69. 10.1186/s12985-019-1182-0 31133031 PMC6537279

[B25] van Doremalen N. Bushmaker T. Morris D. H. Holbrook M. G. Gamble A. Williamson B. N.. (2020). Aerosol and surface stability of SARS-CoV-2 as compared with SARS-CoV-1. N. Engl. J. Med. 382, 1564–1567. 10.1056/NEJMc2004973 32182409 PMC7121658

[B26] Vitchuli N. Shi Q. Nowak J. McCord M. Bourham M. Zhang X. (2010). Electrospun ultrathin nylon fibers for protective applications. J. Appl. Polym. Sci. 116, 2181–2187. 10.1002/app.3182527877442

[B27] Xiong Y. Peng L. (2020). Focusing on health-care providers' experiences in the COVID-19 crisis. Lancet Global Health 8, e740–e741. 10.1016/S2214-109X(20)30214-X 32573442 PMC7190304

[B28] Xu F. Weng B. Materon L. A. Gilkerson R. Lozano K. (2014). Large-scale production of a ternary composite nanofiber membrane for wound dressing applications. J. Bioactive Compatible Polymers 29, 646–660. 10.1177/0883911514556959

[B29] Zhang Y. Z. Su B. Ramakrishna S. Lim C. T. (2008). Chitosan nanofibers from an easily electrospinnable UHMWPEO-doped chitosan solution system. Biomacromolecules 9, 136–141. 10.1021/bm701130e 18078323

